# Argumentation and explanation in the law

**DOI:** 10.3389/frai.2023.1130559

**Published:** 2023-09-04

**Authors:** Antonino Rotolo, Giovanni Sartor

**Affiliations:** ^1^Alma AI and Department of Legal Studies, Alma Mater Studiorum - Università di Bologna, Bologna, Italy; ^2^Department of Law, European University Institute, Florence, Italy

**Keywords:** explanation, argumentation, legal reasoning, defeasibility, normative systems, justification

## Abstract

This article investigates the conceptual connection between argumentation and explanation in the law and provides a formal account of it. To do so, the methods used are conceptual analysis from legal theory and formal argumentation from AI. The contribution and results are twofold. On the one hand, we offer a critical reconstruction of the concept of legal argument, justification, and explanation of decision-making as it has been elaborated in legal theory and, above all, in AI and law. On the other hand, we propose some definitions of explanation in the context of formal legal argumentation, showing a connection between formal justification and explanation. We also investigate the notion of stable normative explanation developed elsewhere in Defeasible Logic and extend some complexity results. Our contribution is thus mainly conceptual, and it is meant to show how notions of explanation from literature on explainable AI and legal theory can be modeled in an argumentation framework with structured arguments.

## 1. Introduction

Argumentation is critically relevant to law, whose application involves deliberation over the ascertainment of uncertain past facts, as well as the interpretation and application of general legal rules to particular cases, in consideration of relevant values and principles.^1^

Legal problem solving involves dialectical and indeed adversarial interactions in which different ways of reasoning are deployed: probabilistic, deductive and presumptive inferences, the use of analogies, appeals to precedent and policy, and the balancing of interests.

Legal decisions have the authority to be coercively enforced, as issuing from the political community. Thus, such decisions need to be justified: reasons must be provided of why certain claims were endorsed, based on what reasons, and it must be specified why such reasons prevailed over the reasons to the contrary. These justifications need to be critically evaluated, to determine whether they succeed in explaining legal decision in a way that is satisfactory for the individuals involved and for the society.

While legal theory has extensively studied legal argumentation (see Perelman and Olbrechts-Tyteca, 1969; MacCormick, 1978; Alexy, 1989), a formal account of it has only been provided by the AI & Law research, which has profited from, and contributed to, the logical tools for argumentation made available within AI (for an overview, see Prakken and Sartor, 2015). In fact, AI & Law researchers have not only applied AI-based argumentation techniques to the law, but have also made innovative contributions to the development of formal models of argumentation.

An open research question, which is drawing more and more attention in the literature, is the conceptual and formal investigation of the relation between justification and explanation of legal decisions, especially, when norms are crucial in the reasoning process. This article will mainly address this issue.

### 1.1. Purpose and synopsis of this contribution

We believe there is a still overlooked research challenge, which, taking stock of major achievements in legal theory, concerns the relationship between the “justification of legal arguments” and the “explanation of normative conclusions.” To tackle this issue, we aim to connect two research domains, employing formal argumentation: the investigation of AI & Law, which focuses on justifying (automated) legal decision-making, and the examination of explanations within the context of eXplainable AI.

Our contribution is primarily conceptual, aiming to demonstrate how ideas proposed in works such as the one by Miller (2019) or explored in legal theory can be represented within an argumentation framework.

In the light of the importance of argumentation for the legal domain, this article thus aims at contributing to the following related aspects:

- Reconstructing, from the AI & Law literature, the main models of legal argument and formal argumentation, and linking these models to the concepts of justification and explanation;
- Given the above conceptual background, proposing some definitions of explanation in the context of formal legal argumentation.

The layout of the article is as follows. Section 2 clarifies the distinction in the law between justification and explanation. Section 3 develops as follows: after recalling why the law is an argumentation framework (Section 3.1), we will discuss the need to provide explanations when norms are used as preconditions for inferring and issuing other norms (Section 3.2) or for applying them (Section 3.3). We will then consider applications in legal interpretation (Section 3.4) and in case-based reasoning (Section 3.5). Sections 4, 5 offer a conceptual analysis of legal explanation in formal argumentation: the building blocks are recalled in Section 4, while Section 5 presents some definitions of the idea explanation in legal argumentation and investigates the concept of stable explanation extending previous work. Section 6 clarifies the originality of our contributions and discusses related and future work. Some conclusions end the paper.

## 2. Justification, explanation, and argumentation in legal reasoning

In this section we shall discuss how argumentation has a foundational role with regard to the justification and explanation of normative conclusions.

### 2.1. Justification and explanation in legal decision-making

An extensive discussion of the relation between normative explanation and justification (Baier, 1958, chap. 6) is beyond the scope of this paper. Let us just remark that, while a vast literature exists on the concept of an explanation in philosophy (Achinstein, 1983; Pitt, 1988) legal theory has mainly focused on justification, taking this concept as central in the context of legal decision-making (Alexy, 1989; Peczenik, 1989). From the legal theory perspective, it may seem that explanations are a byproduct of justifications: the arguments justifying a decision, on the basis of facts and norms, also provide an explanation of the same decision.

The connection between explanation and justification has also emerged in AI, where more attention has been devoted to the concept of explanation, especially in the debate on eXplainable AI (XAI) (Miller et al., 2022). The AI & Law community has also worked toward explanation, since both “*transparency*” and “*justification*” of (automated) legal decision-making require providing explanations (Atkinson et al., 2020; Governatori et al., 2022c; Prakken and Ratsma, 2022).

Legal decision-making (and consequently, also legal advice) is a complex multi-step process that involves addressing factual and normative issues, based on empirical evidence and legal questions. Different answers to such issues are often possible, depending on the ethical and political preferences and the psychological attitudes of the decision-makers. The extent to which such preferences and attitudes may determine the outcome of the case is constrained by the available evidence and applicable norms. However, a space for discretion, broadly understood, remains, and this space is adjustable, since constraints themselves are to be interpreted by the decision-makers, according to their view of the role of decision-maker (typically judges) and of the principle of the separation of powers.

Within an argumentation-based approach, *the justification of a legal decision may be viewed as an argument structure aimed to show that the decision is right or correct, according to a convincing reconstruction of facts and norms*. Justifications are pervasive in the law, since, as noted above, legal decision-makers are usually required to publicly provide rational grounds for the normative correctness of their decisions (at least for important ones). Justifications may also be produced, possibly integrating the original ones, at a subsequent time, by those who agree with such decisions and want to provide further reasons supporting them.

Consider for instance Dobbs v. Jackson Women's Health Organization, 19-1392 U.S. 597 (2022) decision by the US Supreme Court, which denied the existence of a constitutional right to abortion, contrary to the previous Roe v. Wade, 410 U.S. 113 (1973) decision, which had affirmed that right. The majority of the Dobbs judges provided a justification of that decision based on certain legal doctrines on the interpretation of the US constitution (a version of the so-called originalism), on federalism, on the separation of powers, which require according to their view that the legality of abortion is decided at the State level, rather than at the federal level. More extended justifications of that decisions have been provided by legal scholars who agree with its content and want to support its correctness with further considerations. On the other hand, the judges in the dissenting opinion strongly criticized this justification, and so did scholars and activists opposing the Dobbs decision.

In legal theory it is common to distinguish the “discovery” process through which decision-makers endorse certain conclusions on the relevant issues—guided by the information they access, but also by their intuitions and by their tacit expert knowledge—and the process of building an accessible justification of that decision, which may appear convincing or at least acceptable to the parties and the public (MacCormick, 1978). Justification usually follows discovery, and selectively uses the information elicited during discovery, in order to provide a rhetorically effective account. However, dialectical interactions between the two processes exist: on the one hand considerations developed during the discovery process may enter into the justification, on the other hand the necessity to build a convincing justification may guide the process of discovery, leading the decision-makers to reject or amend the outcomes for which a convincing justification could not be found.

It seems to us that in any case a description of the discovery process is no substitute for a justification as just described: first of all, many aspects of the process of discovery are not accessible to description, pertaining to the unconscious working of the decision-maker's mind; secondly, some moves in the discovery process may pertain to taking wrong directions, or anyway to aspects that are not relevant for the goal of providing a publicly acceptable justification. On the other hand, however, certain inference steps that took place during discovery (including logical and statistical inferences, the assessment of competing factors, the interaction of rules and exceptions, presumption, etc.) can be recovered for the purpose of building a justification.

### 2.2. Types of legal explanation: conceptual distinctions

While *justifications are reasoned defenses of (legal) decisions* by the authors of such decisions or third parties supporting the same decisions, *explanations involve a third-party perspective*, which does not presuppose the endorsement of the explained decisions (for a general philosophical discussion, see Davidson, 1963).

We may indeed distinguish two ways of explaining legal decisions: *causal explanations*, and *rational reconstructions*.

Causal explanations of legal decisions aim at identifying social, ideological, or political factors that contribute to the outcomes of legal cases, inducing decision-makers to adopt such outcomes. For instance, in the Dobb case we might consider that the outcome was determined by the political position of the majority of the judges (positioned in the right-wing side and nominated by republican presidents), their religious convictions, their ideological commitments, their connections with certain groups of the population, etc. In some cases, the causal explanation may include pointing to failures in the decisional process: the decision-makers were affected by their prejudices, were bribed, their decision was instrumental to favoring their friends or harm their enemies, etc.

This *extra-legal and extra-systemic explanation* of legal decisions can be distinguished from the *intra-legal and intra-systemic rational explanation* (i.e., a rational reconstruction), by which we may understand the attempt to identify reasons why certain decisions may be legally appropriate, given the beliefs, view-point and political-ethical-legal commitments of those who support such decisions (and first of all of the decision-makers who adopted them). A broad notion, which fits with our analysis, is proposed by Väyrynen (2021) for whom normative explanations are “explanations of why things are wrong, good, or unfair.” In the context of legal decision-making, we may say that a normative explanation is an account of why a legal evaluation (on the legality, illegality of action, the ascription of rights or obligations) is considered to be correct on the basis of *both norms and facts* (a combination that was first emphasized by Schroeder, 2005).

*Rational explanations, as well as justifications can take the shape of an argumentation framework*, in which, besides presenting the arguments favoring the decision, arguments to the contrary are considered and defeated. This perspective involves a “principle of charity,” in the sense that it is assumed that the decision is the outcome of reasoned factual and legal considerations, even though we may disagree with the substance of such considerations. Thus, those who disagree with the Dobbs decision, can still provide a rational explanation (reconstruction) of that decision by presenting a coherent narrative including legally relevant reasons in favor of that decision, together with the assessment of such reasons according to the perspective of those who endorse them. Nevertheless, the opponents of the same decision may continue to consider that it was wrong, since stronger reasons, according to their perspective, exist for reaching the opposite conclusion. The opponent of Dobbs can also merge their critical considerations with the rational explanation of the decision they disagree with. In such a case, a critical argumentation framework is obtained, in which the arguments explaining the decision are defeated by the arguments against that decision (consider for instance, a legal essay that attacks the conclusion as well as the reasoning of the judges in the Dobbs case).

### 2.3. Our conceptual standpoint

In conclusion, it seems to us that both the justification of a legal decision and its rational explanation, as described above, can be captured through argumentation. Both ideas presuppose that an outcome (the decision of a case) should be supported by reasons, and that these reasons should prevail over the reason to the contrary, according to a certain perspective. The *distinction between justification and rational explanation, according to our analysis, pertains to pragmatics, rather than to semantics*. It concerns the purpose of the exercise: providing support to a decision we endorse (being those who propose it, or aim to advance or defend it) or rather accounting for the support that is attributed to a decision by those who endorse it, for reasons endorsed by them. In the following, when speaking of explanations, without further clarifications, we cover both justifications and rational explanations.

The distinction between justification and (rational) explanation thus seems to rely on a perspectival approach. For an agent *a*_1_: (a) a decision *d* (by *a*_1_ or by other agents) is justified iff it is supported by prevailing reasons in the context of the attitudes and beliefs of *a*_1_; (b) a decision *d* by an agent *a*_2_ is (rationally) explained if it is supported by prevailing reasons in the context of the attitudes and beliefs of *a*_2_.

In the context of the application of AI technologies to legal decision-making the relation between discovery (the activity through which a system constructs an answer to a legal issue) and justification-explanation (the attempt to provide reasons for that answer) tends to take a different form in knowledge-based systems (including formal-argumentation system), on the one hand, and in opaque machine learning applications, on the other hand. In the first case, the argumentative justification-explanation of a case can be constructed on the basis of the very inferences and reasoning patterns through which the system came to determine its outcome; in the second case an argumentative justification-explanation has to constructed as a parallel exercise, meant to mimic the opaque inference of the system. In both cases, the construction of argumentative explanations presupposes the availability of a knowledge base of rules and concepts, from which arguments can be constructed.

In this paper, we shall assume that such a knowledge-base is available, and we shall consider to what extent it can be used to build argumentation frameworks. Given an argumentation framework we shall consider, by deploying an argumentation semantics, what arguments and conclusions are supported by that framework, where this notion of support may be viewed as a kind of justification: an outcome is justified by the (grounded) extension or labeling in which it is included. Based on this idea, we shall provide some notions that clarify aspects of legally relevant explanations.

First, we shall discuss whether an explanation can be viewed as an argument set that is suitable to support the explanandum (within the given argumentation framework): if any arguments in the set were not available the explanandum would not be derived, through that explanation.

Focusing especially on factual premises and norms, we shall then consider contrastive explanations, which elicit, under minimality conditions, those facts or norms whose presence or removal would preempt the derivation of the explanandum.

We are aware that our analysis cannot cover all aspects that are addressed under philosophical conceptions of a (normative) explanation, but we believe we will provide a sufficiently rich account that makes an essential use of the distinctive elements the legal knowledge base.

## 3. Models of legal argument

The adoption of argumentative model for the justification-explanation of legal decisions was motivated by the fact that purely deductive approaches fail to capture key aspects of legal reasoning, such as conflicts between competing rules, the relation between rules and exceptions, the significance of factors, interpretive and case-based reasoning, and more generally, the dialectical and adversarial nature of legal interactions (Perelman and Olbrechts-Tyteca, 1969; Alexy, 1989; Walton et al., 2008; Bongiovanni et al., 2018).

Argumentation pervades all the three dimensions of the law distinguished by Hart (1994):

- **Norm recognition and hierarchies:** legal systems consist of norms and provide criteria for establishing whether any norms belong to them; legal systems assign to their norms a different ranking status and organize them in hierarchies (e.g., constitutional norms are stronger than legislative acts);
- **Norm change:** legal systems change and include criteria governing their dynamical evolution;
- **Norm application:** the norms in a legal system are applied to concrete cases, and this process is based on interpretive and procedural criteria specified by that system.

*In a reasoning and argumentative perspective*, we can think of the above dimensions as follows:

- Arguments can be used for inferring, issuing, or adopting norms, and for determining how norms are related with one another (e.g., for establishing when one norm may override another one in case of conflicts; **Norm recognition and hierarchies**);
- Arguments can be used for proposing and implementing revisions to legal systems (**Norm change**);
- Arguments can be used for advancing interpretations of legal provisions, supporting them against alternative interpretations (e.g., when different interpretive canons, as applied to the same provision, offer different legal solutions for the same case) and for applying the resulting norms (**Norm application**).

In the following, we briefly recall the main contribution of argumentation theory in AI & Law to some of these dimensions and identify some challenges to be addressed in regard to the distinction between justification and explanation.

### 3.1. The law as an argumentation framework

It has been argued that the law itself can be described as a complex argumentation framework (Prakken and Sartor, 2015). Under this general assumption, arguments must determine (and thus explain) the way in which *norms interplay with one another in legal systems* (Alchourron and Bulygin, 1971). Defeasible argumentation (Dung, 1995; Pollock, 1995) has indeed been used to address conflicts between norms and ways to resolve such conflicts through meta-arguments, as well as the interactions between legal rules and the reasons supporting them (Hage, 1997; Prakken and Sartor, 2015).

Through formal argumentation, among others, the following challenges can be addressed:

- Explaining the interplay of legal norms. When there is a conflict of legal rules *r*_1_ and *r*_2_, both applicable to the case at hand, then a decision for *r*_1_'s outcome must include a preference for *r*_1_ and possibly reasons for that preference.
- Explaining the application of norms. In deciding a case a procedure has to be followed where facts have be assessed in compliance with legal constraints, rules have to be identified and their applicability assessed.
- Explaining the interpretation of legal norms. When alternative interpretations *i*_1_ and *i*_2_ of a legal provision exist, then a decision for the outcome corresponding to *i*_1_ must be supported by the reasons why *i*_1_ rather than *i*_2_ should be accepted as the interpretation of that provision.

### 3.2. Explaining the interplay of legal norms

Let us first consider the need to provide explanations where norms are used for inferring, issuing, or applying other norms.

Assume that norms in the legal system *L* are represented as rules of the form *r*:*ϕ*_1_, …, *ϕ*_*n*_⇒ψ (where *r* is the name of the norm). Then a preference relation > can capture a hierarchy over *L* that enables collisions between norms being addressed. Consider for example


$$\begin{array}{l} L=\{\{r:\phi_{1},\ldots ,\phi_{n}\Rightarrow \psi ,\;s:\psi \Rightarrow \pi ,\;t:\omega \Rightarrow \neg \pi \}\\ >=\{\langle s,t\rangle \}\}. \end{array}$$

Assume also that the antecedents of *r* and *t*, i.e., facts *ϕ*_1_, …, *ϕ*_*n*_, ω are the case. Because *s* is hierarchically superior to *t*, then an argument *A* concatenating *ϕ*_1_, …, *ϕ*_*n*_, *r* and *s* successfully supports the conclusion π, defeating the argument concatenating ω and *t*. Jurists usually would say that this argument legally grounds and justifies π in *L*. Notice that the law “is not concerned with the absolute rationality of the normative statement in question, but only with showing that it can be rationally justified within the framework of the validly prevailing legal order” (Alexy, 1989, p. 220). This simple context illustrates different legally relevant explanations of π. We may say that conclusion π is explained:

- By argument *A*, which grounds conclusion π upon the relevant facts;
- By the whole of *L* plus all facts of the case, which together provide for the conflicting arguments and for the preference solving their conflict;
- By each fact in *ϕ*_1_, …, *ϕ*_*n*_, since one may counterfactually argue that without any of them we would have ¬π rather than π;
- By each of the rules *r* and *s*, since without either of them π could not be (sceptically) inferred;
- By the preference *s* > *t*, without which also π could not be inferred.

### 3.3. Explaining the application of the law

When the law is applied to cases (e.g., by judges in courts), legal theory traditionally breaks down the analysis of judicial decisions into three dimensions: the so-called question of fact (*quaestio facti*), i.e., reconstructing the facts of the case on the basis of the available evidence, the ways in which proceedings develop (judicial procedures), and the so-called question on law (*quaestio juris*), i.e., interpreting the law to identify the applicable legal rule. Within AI & Law, an in-depth analysis has been developed of evidential reasoning, comparing different approaches to it (Verheij et al., 2016). The procedural aspects of decisions have been investigated in regard to ideas such as the standard of proofs, presumptions, and burdens of proof (Prakken and Sartor, 2006; Calegari and Sartor, 2021; Kampik et al., 2021). Formalizations have also been developed for protocols governing the admissibility and impact of arguments in legal debates (Gordon, 1995; Governatori et al., 2014). More recently, the idea that multiple argument schemes can be used in legal arguments has been explored, as well as the issue of which argumentative strategies are most effective in different legal disputes from a game-theoretical perspective (Roth et al., 2007; Riveret et al., 2008).

Jurists naturally resort to *causal explanation* in the context of reasoning about evidence (Walton, 2002), where competing accounts of the facts of the case are developed on the basis of the available evidence. In this domain, AI & Law research has devoted an extensive effort and discussed classic issues such as the relation between abductive and counterfactual reasoning and legal argumentation (see, again Prakken and Sartor, 2015 for an overview of the literature, see Liepina et al., 2020 for a recent attemp di identify causal argument schemes for causal reasoning).

Logical models have also been used to relate legal norms to the cases at hand and explain why such norms are applicable to the given facts. One framework that has been developed for this purpose is called reason-based logic (RBL), which focuses on how principles, goals, and rules can influence the interpretation of legal provisions (Hage, 1997).

### 3.4. Explaining the interpretation of the law

Legal interpretation has been viewed as a decision-making problem, in which the goal is to choose the best interpretation based on its consequences for promoting and demoting values (Atkinson and Bench-Capon, 2007; da Costa Pereira et al., 2017). Another approach is the argument-scheme approach, which considers interpretive canons using defeasible rules to interpret legal provisions and resolving conflicts by comparing the reasons behind different interpretations (Rotolo et al., 2015; Walton et al., 2021). The latter idea fits legal theories that view interpretive canons as reasoning patterns for constructing arguments aimed at justifying interpretive outcomes. Examples of canons by MacCormick and Summers (1991) are:

**Argument from ordinary meaning**: if a statutory provision can be interpreted according to the meaning a native speaker of a given language would ascribe to it, it should be interpreted in this way, unless there is a reason for a different interpretation.
**Argument by coherence**: a provision should be interpreted in light of the whole statute it is part of, or in light of other provisions it is related to.
**Teleological argument**: a provision should be interpreted as applied to a particular case in a way compatible with the purpose that the provision is supposed to achieve.
**Arguments from general principles**: whenever general principles, including principles of law, are applicable to a provision, one should favor the interpretation that is most in conformity with these general legal principles.

According to Rotolo et al. (2015) and Walton et al. (2021), the structure of interpretive arguments can be analyzed using *interpretation rules*, where the antecedent of interpretation rules can be of any type, while the conclusion is an interpretive act I of a provision *n* leading to an interpretive result ψ for *n* which expresses such an interpretation paraphrasing *n* into ψ. An example of an interpretation rule is the following:


(1) $$\begin{array}{l} r^{\prime }:\phi 1,\ldots ,\phi n\Rightarrow I_{teleological}\left(n_{1}^{L},\psi \right) \end{array}$$

Rule *r*′ states that, if *ϕ*_1_, …, *ϕ*_*n*_ hold, then the interpretive canon to be applied in legal system *L* for provision *n*_1_ is the teleological interpretation, which returns ψ.

Now suppose to have the following rules (the example logically mirrors the one in Section 3.2):


$$\begin{array}{l} R=\{\{r^{\prime }:\phi_{1},\ldots ,\phi_{n}\Rightarrow I_{teleological}(n_{1}^{L},\psi )\\ \;s^{\prime }:I_{teleological}(n_{1}^{L},\psi )\Rightarrow I_{coherence}(n_{2}^{L},\pi ),\\ \;t^{\prime }:\Rightarrow I_{ordinary}(n_{2}^{L},\neg \pi )\}\\ >=\{\langle s^{\prime },t^{\prime }\rangle \}\}. \end{array}$$

In legal theory, we may say that the interpretation of *n*_2_ as π is justified in the legal system *L* (on modeling interpretation through argumentation, see Walton et al., 2021; Sartor, 2023). We may also say that the argument built with *r*′ and *s*′ explains this outcome, or, also, that *ϕ*_1_, …, *ϕ*_*n*_ explain it.

### 3.5. Explaining the use of judicial cases

Legal systems often rely on past cases to guide decision-making and legal reasoning. A popular AI & Law approach to case-based reasoning consists in focusing on factors, namely, on features of cases that favor or disfavor certain outcomes (Rissland and Ashley, 1987; Ashley, 1990; Ashley and Aleven, 1991). The presence or absence of certain factors in a new case, or in precedents cases, can be used to support or challenge legal claims. There have been various developments of the factor-based approach within AI & Law, including the use of multivalued factors (Bench-Capon and Rissland, 2002) and hierarchies of factors (Aleven and Ashley, 1997), as well as logical mechanisms for determining when a decision is consistent or inconsistent with a case base (Horty, 2011).

Investigations have been developed on the combination of models of case-based reasoning with formal approaches to defeasible argumentation (Berman and Hafner, 1993; Bench-Capon and Sartor, 2003; Bench-Capon et al., 2013; Maranhão et al., 2021). Accordingly, a case can be reconstructed as expressing two competing rules and a preference for one of them (Prakken and Sartor, 1998): the conjunction of the factors *ϕ*_1_, …, *ϕ*_*n*_ which are present in the case and support its outcome ψ corresponds to a defeasible rule *ϕ*_1_, …, *ϕ*_*n*_⇒ψ, which prevails over the rule χ_1_, …, χ_*m*_⇒¬ψ, whose antecedent is the conjunction of all factors χ_1_, …, χ_*m*_ in the case which support the outcome ¬ψ. The rules involved in factor-based reasoning are defeasible in that new factors can explain deviations from earlier decisions.

In Liu et al. (2022a) case-based reasoning and classifier systems are connected, and on this basis different kinds of case-based explanations are defined such as abductive and contrastive ones. The logic of Liu et al. (2022a) is based on modal logic and does not directly capture the argumentative nature of case-based reasoning, as recalled above. Prakken and Ratsma (2022) uses argumentation—based on multi-valued factors (dimensions)—to explain the outcome of legal cases.

## 4. Formal argumentation

In this section we present formal argumentation and illustrate its application to legal reasoning. Argumentation frameworks have been proposed by Dung (1995) to investigate the general aspects of dialectical reasoning without specifying the internal structure of arguments. Many semantic models have been developed (Baroni and Giacomin, 2009) for abstract argumentation. Such models determine what arguments can be accepted, by considering not only how such arguments directly conflict with each other, but also how arguments can be indirectly defended by other arguments. Among them, several options have been acknowledged as appropriate in legal reasoning (see Prakken and Sartor, 2023). However, since we work in this paper on argumentation for reasoning with norms, we follow Governatori et al. (2021) and Governatori and Rotolo (2023). These works suggest that when norms collide and no priority principles can apply (such as the principles *lex superior, lex posterior* and *lex specialis*), a skeptical approach may be the most appropriate one, especially when legal effects of norms are obligations or sanctions. For the sake of simplicity, we focus on grounded semantics.

Let us first of all recall from the literature some basic formal concepts.

Definition 1 (**Argumentation framework and semantics**).

***Argumentation framework***. An argumentation framework AF is a pair (A, ≫) where A is a set of arguments, and ≫⊆A×A is a binary, attack relation.
***Conflict-free set***. A set S of arguments is said to be conflict-free if, and only if there are no arguments *A* and *B* in S such that *B* attacks *A*.
***Argument defense***. Let S⊆A. The set S *defends* an argument *A*∈A if, and only if for each argument *B* attacking *A* there is an argument *C*∈S that attacks *B*.
***Complete extension***. Let AF = (A, ≫) and S⊆A. S is a complete extension of AF if and only if S is conflict-free and S = {*A*∈A|Sdefends*A*}.
***Grounded extension***. A grounded extension *GE*(AF) of an argumentation framework AF is the minimal complete extension of AF.
***Justified argument and conclusion***. An argument *A* and its conclusion Conc(*A*) are justified w.r.t. an argumentation framework AF if, and only if *A*∈*GE*(AF).
***Rejected argument and conclusion***. An argument *A* and its conclusion Conc(*A*) are rejected w.r.t. an argument framework AF is, and only if *A*∉*GE*(AF).

While abstract argumentation is not concerned with the internal structure of arguments, it was argued in the AI & Law literature the importance of devising argumentation frameworks where arguments have a logical structure (see Sartor, 2005; Prakken and Sartor, 2015; Governatori et al., 2021). If the underlying language of an argumentation framework refers to any logic ***L***, arguments can roughly correspond to proofs in ***L*** (Prakken and Vreeswijk, 2002). As done by Governatori et al. (2004), Prakken (2010), and Toni (2013), given the above framework the (internal) logical structure of arguments can be specified using rule-based systems in such a way that rules correspond, e.g., to norms or normative reasoning patterns (such as in the case of interpretation rules) (Sartor, 2005; Prakken and Sartor, 2015; Governatori et al., 2021) and arguments are logical inference trees built from them.

Definition 2 (**Language**). The language consists of *literals* and *defeasible rules*. Given a set PROP of propositional atoms, the set of *literals* is Lit = PROP∪{¬*p* | *p*∈PROP}. We denote with ~*ϕ* the *complementary* of literal *ϕ*; if *ϕ* is a positive literal ψ, then ~*ϕ* is ¬ψ, and if *ϕ* is a negative literal ¬ψ, then ~*ϕ* is ψ.

Let Lab be a set of unique rule labels. A *defeasible rule* *r* with *r*∈Lab has the form Ant(*r*)⇒Head(*r*), where

- Ant(*r*), called the *antecedent* or the *premises* of *r*, is a subset of Lit (which may be empty) and
- Head(*r*) is a literal in Lit, called the *consequent* or *head* of *r*.

If *R* is a set of rules,

- *R*[*ϕ*] is the set of rules in *R* with head *ϕ*,
- ANT(*R*) is the union of all antecedents of all rules in *R* (i.e., it contains all literals in the antecedents of such rules).

Any defeasible rule whose antecedent is satisfied provides sufficient support to its conclusion unless there is evidence contrary to that conclusion.^2^

Following Governatori et al. (2004) we use the term argumentation theory to denote the rule-based knowledge from which argumentation frameworks are built. Notice that, as done by Antoniou et al. (2001), we distinguish a set of indisputable statements called *facts*, even though, without loss of generality, we impose some restrictions on it to keep things simpler.

Definition 3 (**Argumentation theory**). An *argumentation theory* *D* is a structure


(R,F,>)

where

- *R* is a (finite) set of defeasible rules,
- *F* ⊆ Lit is a consistent set of indisputable statements called *facts* such that, for each φ∈*F*, *R*[φ]∪*R*[*~φ*]=∅, and
- >⊆*R*×*R* is a binary relation on *R* called *superiority relation*.

The relation > describes the relative strength of rules, that is to say, when a single rule may override the conclusion of another rule; it is required to be irreflexive, asymmetric and acyclic (i.e., its transitive closure is irreflexive).

By combining the rules in a theory, we can build arguments [we adjust the definition by Prakken (2010) to meet Definition 3]. Let us first introduce some notation: for a given argument *A*, Conc(*A*) returns *A*'s conclusion, Sub(*A*) returns all its sub-arguments, Rules(*A*) returns all the rules in the argument and, finally, TopRule(*A*) returns the last inference rule in *A*.

Definition 4 (**Argument**). Let *D* = (*R, F*, >) be an argumentation theory. An argument *A* for *ϕ* constructed from *D* has either the form ⇒_*F*_*ϕ* (*factual argument*), where *ϕ*∈*F*, or the form *A*_1_, …, *A*_*n*_⇒_*r*_*ϕ* (*plain argument*), where 1 ≤ *k* ≤ *n*, and

- *A*_*k*_ is an argument constructed from *D*, and
- *r*:Conc(*A*_1_), …,Conc(*A*_*n*_)⇒*ϕ* is a rule in *R*.

With regard to a factual argument ⇒_*F*_*ϕ*:


$$\begin{array}{l} Conc\left(A\right)=\phi \emptyset ;\;Sub\left(A\right)=\emptyset ;\;TopRule\left(A\right)=\\ \emptyset ;\;Rules\left(A\right)= \end{array}$$

With regard to a plain argument *A* = *A*_1_, …, *A*_*n*_⇒_*r*_*ϕ*:


$$\begin{array}{l} Conc\left(A\right)=\phi \\ Sub\left(A\right)=Sub\left(A_{1}\right),\ldots Sub\left(A_{n}\right),A\\ TopRule\left(A\right)=r:Conc\left(A_{1}\right),\ldots ,Conc\left(A_{n}\right)\Rightarrow_{r}\phi \\ Rules\left(A\right)=Rules\left(A_{1}\right),\ldots ,Rules\left(A_{n}\right),TopRule\left(A\right). \end{array}$$

We only consider conflicts between arguments *A* and *B* such that the conclusion of *A* contradicts the conclusion of a subargument *B*′ of *B*.

Conflicts between arguments having contradictory conclusions are resolved on the basis of a last-link ordering. An argument *A* is stronger than another argument *B* (*A*>*B*) if, and only if TopRule(*A*) is stronger than TopRule(*B*) [TopRule(*A*)>TopRule(*B*)]. Notice that we do not need to consider conflicts involving arguments of the form ⇒_*F*_*ϕ* since the set of facts is assumed to be consistent and no fact (or its negation) can occur in the head of any rule.^3^

Definition 5 (**Defeats**). An argument *B* *defeats* an argument *A* if, and only if ∃*A*′∈Sub(*A*) such that Conc(*B*) = ~Conc(*A*′), and *A*′≯*B*.

An argument *B* *strictly defeats* an argument *A* if, and only if *B* defeats *A* and *A* does not defeat *B*.

We can now define the argumentation framework that is determined by an argumentation theory.

Definition 6. (**Argumentation framework in structured argumentation**). Let *D* = (*R, F*, >) be an argumentation theory. The *argumentation framework* AF(*D*) *determined by* *D* is (A, ≫) where A is the set of all arguments constructible from *D*, and ≫ is the defeat relation defined above.

Given this definition of argumentation framework, if *D* is an argumentation theory, we can abuse notation somewhat and write *GE*(*D*) to denote the grounded extension of the argumentation framework determined by *D*.

As noted above we consider that an argument is justified iff it is included in the grounded extension, and a conclusion justified iff it is supported by a justified argument.

Example 1. Consider the following theory *D*, describing a COVID scenario adapted from Italian temporary legal measures to prevent the spreading of pandemics (Governatori et al., 2022a).^4^


$$\begin{array}{l} F=\{positive,vax,\neg mask,old\}\\ R=\{r_{1}:positive,quarantine\Rightarrow \neg spread,\\ \;r_{2}:positive\Rightarrow spread,\\ \;r_{3}:positive,mask\Rightarrow \neg spread,\\ \;r_{4}:spread,vax\Rightarrow \neg high\_lethality,\\ \;r_{5}:spread,old\Rightarrow high\_lethality,\\ \;r_{6}:high\_lethality\Rightarrow hospital\_collapse,\\ \;r_{7}:positive\Rightarrow mask\_obligatory,\\ \;r_{8}:hospital\_collapse\Rightarrow lockdown\_obligatory\}\\ >=\{\langle r_{1},r_{2}\rangle ,\langle r_{3},r_{2}\rangle ,\langle r_{5},r_{4}\rangle \}. \end{array}$$

Let us define the set A of arguments from *D*:


$$\begin{array}{l} A=\{A_{1}:\;\Rightarrow_{F}positive,\\ \;A_{2}:\;\Rightarrow_{F}vax,\\ \;A_{3}:\;\Rightarrow_{F}\neg mask,\\ \;A_{4}:\;\Rightarrow_{F}old,\\ \;A_{5}:\;A_{1}\Rightarrow_{r_{2}}spread,\\ \;A_{6}:\;A_{5},A_{2}\Rightarrow_{r_{4}}\neg high\_lethality,\\ \;A_{7}:\;A_{5},A_{4}\Rightarrow_{r_{5}}high\_lethality,\\ \;A_{8}:\;A_{7}\Rightarrow_{r_{6}}hospital\_collapse,\\ \;A_{9}:\;A_{1}\Rightarrow_{r_{7}}mask\_obligatory,\\ \;A_{10}:\;A_{8}\Rightarrow_{r_{8}}lockdown\_obligatory\}. \end{array}$$

The argumentation framework determined by *D* is thus AF(*D*) = (A, ≫) where


$$\begin{array}{l} \gg =\left\{\langle A_{7},A_{6}\rangle \right\}. \end{array}$$

The grounded extension of AF(*D*) is {*A*_1_, *A*_2_, *A*_3_, *A*_4_, *A*_5_, *A*_7_, *A*_8_, *A*_9_, *A*_10_}. The set *GE*(*D*) of justified conclusions is


$$\begin{array}{l} GE(D)=\{positive,\;vax,\;\neg mask,\;old,\;spread,\;high\_lethality,\\ \;hospital\_collapse,mask\_obligatory,\;lockdown\_obligatory\} \end{array}$$

## 5. Types of explanation in legal argumentation

As informally discussed in Sections 2, 3, an open research issue concerns the relation between the *justification of arguments* and the *explanation of legal conclusions*. To address this issue, we shall try to build a bridge between two research lines using formal argumentation: the AI & Law investigation on the justification of (automated) legal decision-making, and the study of the idea of explanation in the context of eXplainable AI. The following sections provide some general ideas to fill the gap and aim at potentially addressing, at an abstract level, the challenges discussed in Sections 2, 3.

In rule-based systems, finding an explanation for a certain normative conclusion *ϕ* (such as a legal conclusion) requires determining if certain pieces of information support the conclusion of *ϕ* through a set of rules (Governatori et al., 2022b). In the context of argumentation, such an intuition should be adjusted and further elaborated. Notice that, in contrast with the majority of the literature (see Section 6) we provide several definitions of the idea of legal explanation that do not simply focus on arguments, but also that make an essential use of the distinctive elements (facts, rules, priorities) of argumentation frameworks.

Our contribution is mainly conceptual and it is meant to show how notions such as those proposed by Miller (2019) or discussed in legal theory can be modeled in an argumentation framework: an extensive formal study is left to future research.

### 5.1. Explanations by sufficient or necessary arguments

Let us first introduce two auxiliary notions, i.e., closure under subarguments and superarguments.

Definition 7. (**Closure under subarguments and under superarguments**). A set *S* of arguments is closed under subarguments iff for every arguments *A*∈*S*, Sub(*A*)⊆*S*.

A set *S* of arguments is closed under superarguments w.r.t. an argument set *W*, iff for every arguments *A*∈*W* and *A*′∈*S* such that *A*′∈Sub(*A*), *A*∈*S*.

Let us begin with two basic concepts of legal explanation that draw inspiration from Hart and Honoré's (1959) NESS theory of legal causation, and which are reframed here to cover arguments built using norms.

We start with the concept of *explanation by sufficient arguments*, by which we mean a minimal set of arguments which, within the given argumentation framework, is sufficient to determine a certain legal outcome.

Definition 8 (**Explanation by sufficient arguments**). Let *D* = (*R, F*, >) be an argumentation theory and AF(*D*) = (A, ≫) be the argumentation framework determined by *D*. The set E⊆A is an *explanation of* *ϕ* *by sufficient arguments w.r.t*. *D* iff

- *A*∈E is an argument for *ϕ* and *A* is justified w.r.t. *D*;
- E is a minimal set such that, for every argument *B*∈A that defeats *A*, there is an argument *C*∈E that strictly defeats *B*;
- E is closed under subarguments.

Notice that a broader concept of explanation by sufficient arguments for a conclusion *ϕ* could be obtained by the set-theoretical union of all explanations by sufficiency of *ϕ*.

Remark 1. The idea of explanation by sufficient arguments may be philosophically linked to Hart and Honoré's (1959) NESS approach to causality, where a cause for an effect is a necessary element of a sufficient set of conditions for that effect. In our framework, any explanation by sufficient arguments E of *ϕ* is a sufficient set for *ϕ*.

Within formal argumentation, the idea of an explanation by sufficient arguments has been firstly elaborated with minor differences by Fan and Toni (2015) with the idea of related admissibility, which states that a set of arguments E is relatedly admissible iff ∃*A*∈E s.t. E defends *A* and E is admissible. In particular, the authors identify a case where E is minimal (they call this case *minimal explanation*). A difference with respect to our definition is that we focus on the conclusion *ϕ* (which can be supported by more than one argument) and not on a single argument. A similar analysis has been also proposed by Borg and Bex (2020).

The second notion of explanation of a proposition is that of *explanation by necessary arguments*. This includes a set of arguments such that their omission from the argumentation framework would prevent the proposition being justified. Note that this notion is independent from the notion of explanation by sufficient arguments, as introduced in Definition 8.

Definition 9 (**Explanation by necessary arguments**). Let *D* = (*R, F*, >) be an argumentation theory and AF(*D*) = (A, ≫) be the argumentation framework determined by *D*, and *ϕ* be a justified conclusion of AF(*D*). The set E⊆A is an *explanation by necessary arguments* of *ϕ* w.r.t. AF(*D*) iff

- *ϕ* is not justified w.r.t. AF′(*D*′) = (A/*S*, ≫′), where *S* is the closure under superarguments of E relatively to A and ≫′ = ≫−{〈*A, B*〉|*A*∈*S* or *B*∈*S*};
- E is minimal.

Example 2. According to Definition 9, assume that AF(*D*) contains argument [[*a*]⇒*b*]⇒*c* as well as argument [*d*]⇒*c*]. Then *c* is explained through necessary arguments by any set including a subargument for each of these arguments. For instance *c* is explained by {[*a*]⇒*b*], *d*}}, since *c* cannot be established if both [*a*]⇒*b*] and *d* were not available.

Remark 2. Notice that Borg and Bex (2020) have also considered the explanation by necessary arguments. In this work, however, the focus *X* is on single arguments and the target (i.e., the *Y* for which *X* is necessary) is an argument and not a legal conclusion *ϕ* (a conclusion can in fact be supported by more arguments). For this reason, the authors do not explicitly state that, when considered more necessary arguments, *S* must be closed under superarguments.

In legal reasoning often the rules are assumed to be fixed and we only consider the facts as relevant explanations. For instance, if asked why one got a fine, a sufficient answer may consist in pointing to the fact that the speed was 100 km per hour, if it is fixed the set of norms containing the rule prohibiting such a speed.

Following this idea, we can provide the following notions of explanations by sufficient and necessary facts, extracting factual arguments from explanations by sufficient and necessary arguments.

Definition 10 (**Explanation by sufficient/necessary facts**). Let D = (*R, F*, >) be an argumentation theory and AF(*D*) = (A, ≫) be the argumentation framework determined by *D*. The set F is an *explanation of* *ϕ* *by sufficient/necessary facts w.r.t*. *D* iff

- E is and explanation by sufficient/necessary arguments of *ϕ* and
- F is the set of all and only the factual arguments in E.

### 5.2. Contrastive explanations

Let us now consider some specifications of an idea of explanation that is well-known in the literature (Miller, 2019), which is widely used in XAI (Miller et al., 2022), and which has been recently considered in the context of legal reasoning (Borg and Bex, 2020; Liu et al., 2022a). We may informally characterize such explanations as follows:

Intuition 1 (**Contrastive explanation**). Saying that *ϕ* is contrastively explained by *x*′ means saying that if *x*′ rather than *x* had been the case, then *ϕ*′ rather than *ϕ* would have been the case.

We may develop the intuition above depending on whether we consider facts or rules. Indeed, the idea for modeling such a notion is to remove/add relevant facts or rules in such a way that the justification status of *ϕ* will change, and use these changes to provide (part of) an explanation (see Liu et al., 2022b, following Miller, 2019).

Note that our notion of a constrastive explanation covers two different ways in which the justification of a proposition can be interfered with. The inteference may consist in (a) removing from the theory elements being used in arguments that directly or indirectly support the proposition at stake or (b) inserting in the theory elements to be used in arguments that directly or indirectly attack the proposition at stake. Obviously, indirect support consists in attacking attackers and indirect attack in attacking defenders.

Let us first focus on the facts (the literals) that are being used to build legal arguments. We then consider what arguments would be available if the set of facts were changed, adding and/or removing some facts. Thus the contrastive explanation is obtained by considering a minimal pair 〈*F*^−^, *F*^+^〉 where *F*^−^ are the facts to be deleted, and *F*^+^ the facts to be consistently added (i.e., such that *F*∪*F*^+^ is consistent).

Definition 11 (**Fact-based contrastive explanation**). Let *D* = (*R, F*, >) be an argumentation theory and *ϕ* be justified w.r.t. *D*. Then 〈*F*^−^, *F*^+^〉, is a *fact-based contrastive explanation of* *ϕ* *w.r.t*. AF(*D*) iff

1. (*F*\*F*^−^)∪*F*^+^ is consistent;
2. *ϕ* is not justified w.r.t. *D*′ = (*R*, (*F*\*F*^−^)∪*F*^+^), >);
3. no 〈*F*^′−^ ⊆ *F*^−^, *F*^′+^⊆*F*^+^〉, where *F*^′−^∪*F*^′+^⊂*F*^+^∪*F*^−^, satisfies conditions 1 and 2.

Example 3. Let us apply Definition 11 to Example 1 above. It appears that a fact-based contrastive explanation for *lockdown*_*obligatory* is provided by 〈{*positive*}, ∅〉: *positive* contrastively explains that outcome since, without this fact the explanandum would not be justified (if positivity were not the case there would be no obligatory lockdown). Another explanation for the same explanandum would be 〈{*old*}, ∅〉.

Similarly, 〈{¬*mask*}, {*mask*}〉 is an explanation for *lockdown*_*obligatory*, since if people had masks rather than not having them, the explanandum would not hold. In fact, under such a change, all the rest remaining the same, we can infer ¬*spread* so defeating the argument for spread. This would prevent the derivation of *high*_*letality*, *hospital*_*collapse* and *lockdown*_*obligatory*.

Besides contrastively explaining a proposition *ϕ*, as in Definition 11, we may also contrastively explain the non-acceptance of a proposition relative to a theory, i.e., of the failure to provide a justification for it.

Definition 12. (**Fact-based contrastive explanation of non-acceptance**). Let *D* = (*R, F*, >) be an argumentation theory and *ϕ* not be justified w.r.t. *D*. Then 〈*F*^−^, *F*^+^〉, is a *fact-based contrastive explanation of the non-acceptance of* *ϕ* *w.r.t*. AF(*D*) iff

1. (*F*\*F*^−^)∪*F*^+^ is consistent;
2. *ϕ* is justified w.r.t. *D*′ = (*R*, (*F*\*F*^−^)∪*F*^+^), >);
3. no 〈*F*^′−^⊆*F*^−^, *F*^′+^⊆*F*^+^〉, where *F*^′−^∪*F*^′+^⊂*F*^+^∪*F*^−^, satisfies conditions 1 and 2.

Example 4. Consider again Example 1 add to it the following rule, according which if the pandemic does not spread, we can have a normal life under the pandemic:


$$\begin{array}{ll} r_{9}: & \neg spread\Rightarrow normal\text{\_}life \end{array}$$

We may than ask “Why is it that we cannot have a normal life,” and an answer would be the contrastive explanation 〈{¬*mask*}, {*mask*}〉: people are not wearing masks (rather than wearing them). In fact, after the theory is revised by removing, ¬*mask* and adding *mask*, there is a justified argument for *normal*_*life*, based on rule *r*_9_, whose antecedent condition ¬*spread* can be establishes by using rule *r*_3_, and facts *positive*, and *mask*.

The ideas just described can be expanded by assuming that also rules can be removed or added. The rules to be removed are included in the current theory, while the rules to be added can be built from the language (see Definition 2). Thus we obtain the following definition, which matches Definition 11 above.

Definition 13 (**Rule-based contrastive explanation**). Let *D* = (*R, F*, >) be an argumentation theory and *ϕ* be justified w.r.t. *D*. Then 〈*R*^−^, *R*^+^〉, with , *R*^−^⊆*R* and *R*^+^⊆Rul, is a *rule-based contrastive explanation of* *ϕ* *w.r.t*. AF(*D*) iff

1. *D*′ = (*R*\*R*^−^)∪*R*^+^, *F*, >′) where >′ = >−{〈*r, r*′〉|{*r, r*′}∩*R*^−^≠ };
2. *ϕ* is not justified w.r.t. *D*′;
3. no 〈*R*^′−^, *R*^′+^〉, such that (*R*^′−^∪*R*^′+^)⊂(*R*^−^∪*R*^+^), satisfies conditions 1 and 2.

Finally, by combining the possibility to add or remove facts, rules, or even rule-priorities, we come to the following definition:

Definition 14. (**Fact-, rule-, and priority-based contrastive explanation**). Let *D* = (*R, F*, >) be an argumentation theory, AF(*D*) = (A, ≫) be the argumentation framework determined by *D*, and *ϕ* be justified w.r.t. *D*. Then 〈*F*^−^, *F*^+^〉, 〈*R*^−^, *R*^+^〉, 〈>^−^, >^+^〉, with *F*^−^, *F*^+^⊆ANT(*R*), *R*^−^, *R*^+^⊆Rul, >^−^, >^+^⊆Rul × Rul is a *fact-rule-priority-based contrastive explanation of* *ϕ* *w.r.t*. AF(*D*) iff

1. AF(*D*′) = (A, ≫) is the argumentation framework determined by *D*′ = (*R*\*R*^−^)∪*R*^+^, *F*\*F*^−^)∪*F*^+^, (>\>^−^)∪>^+^))
2. *ϕ* is not justified wrt *D*′;
3. Conditions 1 and 2. are satisfied by no triplet 〈*F*^′−^, *F*^′+^〉, 〈*R*^′−^, *R*^′+^〉, 〈>^′−^, >^′+^〉, such that ∪(*F*^′−^, *F*^′+^, *R*^′−^, *R*^′+^, >^′−^, >^′+^)⊂∪(*F*^−^, *F*^+^, *R*^−^, *R*^+^, >^−^, >^+^).

The definitions above are abstract and fit the structure of argumentation frameworks: the effective process of defining minimal revisions of rules and priorities is rather complex (see Billington et al., 1999; Governatori and Rotolo, 2010; Boella et al., 2016; Governatori et al., 2019).

Example 5. Consider again Example 1 and the normative conclusion *lockdown*_*obligatory*. Trivially, 〈{*r*_2_}, ∅〉, 〈{*r*_5_}, ∅〉, 〈{*r*_6_}, 〉, and 〈{*r*_8_}, ∅〉 are rule-based contrastive explanations of *lockdown*_*obligatory* w.r.t. AF(*D*).

Assume that *F* would already include the fact *countryside* and suppose to change the theory *D* into *D*′ = (*R*′, *F*, >) as follows:


$$\begin{array}{ll} R^{\prime }= & R\cup \left\{r_{9}:countryside,spread\Rightarrow \neg lockdown\text{\_}obligatory\right\}. \end{array}$$

Then, we would have two new arguments


$$\begin{array}{l} A_{12}:\Rightarrow_{F}countryside,\\ A_{13}:A_{5},A_{12}\Rightarrow_{r_{9}}\neg lockdown\text{\_}obligatory \end{array}$$

Since we work in a skeptical semantics, 〈{*r*_9_}, ∅〉 is a rule-based contrastive explanation of *lockdown*_*obligatory* w.r.t. AF(*D*).

Finally, suppose we obtain *D*′ by simply making > empty: then, 〈∅,∅〉, 〈∅,∅,〈>^−^, ∅〉 is a fact-rule-priority-based contrastive explanation of *lockdown*_*obligatory* w.r.t. AF(*D*).

### 5.3. Discussion and further examples

Contrastive explanation is perhaps the best example to highlight the third-party nature of explanations as discussed in Section 2. Indeed, such a type of explanation explicitly compares two different argumentation theories and frameworks, which could in fact correspond to two different argumentative angles: one could be attributed to the decision-maker and one of to any observer that rationally reconstructs the decision and explains it by comparison.

More precisely, the actual argumentation framework where we justify a certain legal conclusion provides the perspective of the decision-maker D, while the comparison between this framework and anything else is made by a neutral observer O.

Example 6. Let us go back to the case of legal interpretation briefly recalled in Section 3.4 and consider the following provision from the Italian penal code:

Art. 575. Homicide. Whoever causes the death of a *man*

[*uomo*] is punishable by no less than 21 years in prison.

The almost unanimous interpretation of courts of art. 575 is that, of course, it covers killing of *any* person and not only of men. For doing so, one may consider the ordinary interpretation of art. 3 of the Italian constitution, which establishes, among other things, that all people have equal social status and are equal before the law, without regard to any personal aspects including gender. This is an argument from general principles. Alternatively, one may use an argument by coherence and maintain that the ordinary interpretation of other legislative provisions *n* does the same. Both exclude the ordinary reading of “man” as “adult male human being.” Consider the following argumentation theory *D*, where ψ means “only the death of a human male is punishable by no less that 21 years of prison”:


$$\begin{array}{l} R=\{r^{\prime }:I_{ordinary}(art.3,\pi )\Rightarrow I_{constitutional\_principle}(art.575,\neg \psi )\\ \;s^{\prime }:I_{ordinary}(n,\gamma )\Rightarrow I_{coherence}(art.575,\neg \psi ),\\ \;t^{\prime }:\Rightarrow I_{ordinary}(art.575,\psi )\\ \;z^{\prime }:I_{constitutional\_principle}(art.575,\neg \psi )\Rightarrow \neg \psi ,\\ \;z^{\prime \prime }:I_{coherence}(art.575,\neg \psi )\Rightarrow \neg \psi ,\\ \;z^{\prime \prime \prime }:I_{ordinary}(art.575,\psi )\Rightarrow \psi \}\\ F=\{I_{ordinary}(art.3,\pi ),I_{ordinary}(n,\gamma )\}\\ >=\{\langle z^{\prime },z^{\prime \prime \prime }\rangle \}\}. \end{array}$$

Suppose a court decides a case rejecting ψ and supports ¬ψ because of *r*′, i.e., in the light of art. 3. Indeed, since A in AF(*D*) includes


$$\begin{array}{l} A_{1}:\Rightarrow_{F}I_{ordinary}\left(art.3,\pi \right)\\ A_{2}:A_{1}\Rightarrow_{r^{\prime }}I_{constitutional\text{\_}principle}\left(art.575,\neg \psi \right)\\ A_{3}:A_{2}\Rightarrow_{z^{\prime }}\neg \psi \end{array}$$

then the argument *A*_3_ and its conclusion ¬ψ using *r*′ and *z*′ are justified in the corresponding argumentation framework AF(*D*).

Preliminarily, we should note that

- {*A*_3_, *A*_2_, *A*_1_} is an explanation by sufficient arguments of ¬ψ;
- {*A*_3_, *A*_2_, *A*_1_} is not an explanation by necessary arguments of ¬ψ if we added the rules

$$\begin{array}{l} w:I_{principle}\left(art.575,\neg \psi \right)\Rightarrow I_{teleological}\left(art.575,\neg \psi \right),\\ w^{\prime }:I_{teleological}\left(art.575,\neg \psi \right)\Rightarrow \neg \psi , \end{array}$$

and changed the priorities as follows

$$\begin{array}{l} >=\left\{\langle z^{\prime },z^{\prime \prime \prime }\rangle ,\langle w^{\prime },z^{\prime \prime \prime }\rangle \right\} \end{array}$$

being still {*A*_3_, *A*_2_, *A*_1_} an explanation by sufficiency.

This could be enough in the perspective of the decision-maker D. Let us rationally reconstruct D's decision. Such a reconstruction may correspond to an observer O: several options are available. Let us see three of them for the sake of illustration.

1. If $F^{-}=\left\{I_{ordinary}\left(art.3,\pi \right)\right\}$ then 〈∅, *F*^−^〉 is a fact-based contrastive explanation of ψ: O's explanation of D's decision in favor of ψ is based on noticing that this fact, if removed, would prevent the conclusion.
2. Since A in AF(*D*) includes the following set of justified arguments

$$\begin{array}{l} A_{1}:\Rightarrow_{F}I_{ordinary}\left(art.3,\pi \right)\\ A_{2}:A_{1}\Rightarrow_{r^{\prime }}I_{constitutional\text{\_}principle}\left(art.575,\neg \psi \right)\\ A_{3}:A_{2}\Rightarrow_{z^{\prime }}\neg \psi \\ A_{4}:\Rightarrow_{F}I_{ordinary}\left(n,\gamma \right)\\ A_{5}:A_{4}\Rightarrow_{s^{\prime }}I_{coherence}\left(art.575,\neg \psi \right)\\ A_{6}:A_{5}\Rightarrow_{z^{\prime \prime }}\neg \psi \end{array}$$

while D could only explicitly rely on *A*_3_, the observer O would contrastively explain the decision by noticing that 〈{*r*′, *s*′}∅, 〉 is rule-based contrastive explanation of ¬ψ.
Finally, assume to change the argumentation theory in such a way that >= ∅. Then D would not decide in favor of ¬ψ. Since we work in skeptical argumentation, an observer O can explain this decision by identifying elements that would be needed to conclude ¬ψ and by simply noting that

$$\langle \emptyset ,\emptyset \rangle ,\langle \{r^{\prime },s^{\prime }\},\emptyset \rangle ,\langle \emptyset ,\{\langle z^{\prime },z^{'''}\rangle \}\rangle$$

is a fact-rule-priority-based contrastive explanation of ¬ψ.

### 5.4. Stable argumentative explanations

An interesting issue for investigation is the concept of *stable explanation* in argumentation, a concept that was explored from a proof-theoretic perspective, among others, by Brewka et al. (2019); Brewka and Ulbricht (2019); Governatori et al. (2022b). In particular, Governatori et al. (2022b,c) considered the problem of determining a stable normative explanation for a certain legal conclusion, which means to identify a set of facts (i.e., reasoning inputs) able to ensure that such a conclusion continues to hold when new facts are added to a normative case. The basic intuition is the following.

Intuition 2 (**Stable explanation**). A normative explanation for a given legal conclusion *ϕ* is stable when adding new normative elements to that explanation does not affect its power to explain *ϕ*.

Interestingly, in the context of legal argumentation, we can observe the following (Governatori et al., 2022c):

- Given the facts of the normative case, any judicial proceeding has the objective of determining what *legal requirements* (e.g., obligations, prohibitions, permissions, ascription of rights) hold, and whether such legal requirements have been fulfilled;
- If new facts were presented by one party in the proceeding, the outcome of the case could change;
- Each party in the judicial proceeding is thus interested in the following question: *How to ensure a specific outcome for a case, which, in an adversarial context, means how to ensure that the facts presented by such a party are “resilient” to the attacks of the opponent*?

The following example is adapted from Australian commercial law and from Governatori et al. (2022b,c), and illustrates the idea.^5^

Example 7. Suppose the law forbids private individuals engaging in credit activities. However, such activities are permitted if you have a credit license. Moreover, they are also permitted if you are acting on behalf of another person (the principal), who holds a credit license. In any case, such activities are prohibited if you have been banned from them by the competent regulatory authority. Consider the following theory *D*:


$$\begin{array}{l} F=\emptyset \\ R=\{s_{1}:creditActivity\Rightarrow violation,\\ \;s_{2}:creditLicense,creditActivity\Rightarrow \neg violation,\\ \;s_{3}:actsOnBehalfPrincipal,principalCreditLicense,\\ \;creditAcivity\Rightarrow \neg violation,\\ \;s_{4}:banned,creditActivity\Rightarrow violation\}\\ >=\{\langle s_{2}>s_{1}\rangle ,\langle s_{3}>s_{1}\rangle ,\langle s_{4}>s_{2}\rangle ,\langle s_{4}>s_{3}\rangle \}. \end{array}$$

It is easy to see that relative to theory *D* we can distinguish stable and unstable explanations:

- *F*∪{*creditActivity*} is not a stable explanation for *violation* w.r.t. *D*, since it is no explanation for *violation* in *D*′ if facts are {*creditActivity, creditLicense*} (*violation* not being a justified conclusion w.r.t. *D*′):
- *F*∪{*banned, creditActivity*} is stable explanation for *violation* w.r.t. *D*, since there are no facts *F*′ consistent with *F* (and with the conclusions of the rules in *R*) such that *F* is not an explanation of *violation* with regard to *D*′= (*R, F*∪*F*′, >).

Here is a definition of a stable normative explanation, based on the analysis just provided. In the context of stable explanation by sufficient facts we need to consider facts that (a) are additional to the facts in the theory (b) are consistent with the such facts.

Definition 15 (**Stable explanation by sufficient facts**). Let *D* = (*R, F*, >) be an argumentation theory and F be the set of factual arguments of AF(*D*). An explanation E′⊆F by sufficient facts is *stable relative to* *D* if there is no set of facts *F*′ such that

- *F*∩*F*′=,
- *F*′ is consistent with *F*, and
- E′ is not an explanation by sufficient facts relative to *D*′ = (*R, F*∪*F*′, >).

It is easy to check that this definition works relative to the examples above. For instance, $ℰ\prime =\{\Rightarrow_{F}creditActivity\}$ is no stable explanation by sufficient facts of *violation*, since adding {*creditLicense*} to the facts is such that there is no explanation of *violation* relative to the facts {*creditActivity, creditLicense*}.

A broader account of Governatori et al. (2022b)'s approach is rule-based and proof-theoretic (in Defeasible Logic: Antoniou et al., 2001) while a deontic extension of it has been developed by Governatori et al. (2022c) to characterize the idea of deontic explanation. Relative to an argumentation setting such as the one from Section 4, we can establish the following theorem (for the proof, see Appendix).

Theorem 1. Given a theory *D* and an explanation by sufficient facts F relative to *D*, the problem of determining if F is stable is co-NP-complete.

## 6. Related and future work

We have provided multiple characterisations for the idea of normative explanation in legal argumentation. We hope that our work, though coherent with previous literature, may contribute to further developments on the interaction between argumentation and explanation in the legal domain. The following lines of inquiry are especially relevant to our endeavor:

- Research on explanation in argumentation;
- Research on explanation in the AI & Law domain;
- Research on norm revision and other issues in legal reasoning.

### 6.1. Explanation in argumentation

The idea of modeling explanations in an argumentation framework for decision-making is not new (for an overview, see Cyras et al., 2021b). Approaches to argument-based decision-making have been developed, where argumentation is used to evaluate arguments for and against potential decisions, with the argumentation frameworks constituting the explanations (Amgoud and Prade, 2009). Our approach is connected to this idea, though we extract explanations from argumentation frameworks, rather than viewing argumentation framework as explanations.

The goal of providing explanation through argumentation has inspired the research by Toni et al. starting from (Fan and Toni, 2015) [several subsequent contributions appeared and recent developments have been proposed in several applied fields such as medical diagnostics (Cyras et al., 2021a)]. They construct arguments using rules as we do and elaborate the idea of explanation in an argument-based way (also considered in Cyras et al., 2021b). They sees *explanation of an argument* *A* as a relation between *A* and a subset E of a set of admissible set of arguments to which *A* belongs. Different appropriateness criteria are adopted to define E, according to which explanations can be classified into different types: minimal explanation, compact explanation, maximal explanation, and so forth. Differently from them we have focused the need to provide an appropriate *explanation for a legal conclusion*, i.e., and explanation that may be meaningful for the humans involved (relying on Miller, 2019), thus focusing particularly on contrastive explanations. Our work is also related to Borg and Bex (2021a,b), who propose similar definitions of explanation by sufficient and necessary arguments, but who do not consider several contrastive models.

Other relevant contributions in decision-making are Liao and van der Torre (2020) and Besnard et al. (2022), which however reconstruct explanations within an abstract argumentation perspective.

### 6.2. Explanation in legal argumentation and AI & Law

The concept of explanation has played an important role in the AI & Law community, being related with the general quest of justification and transparency of legal decision-making (Atkinson et al., 2020). Within this community, argument-based explanations have been considered in the domain of evidence (Walton, 2005; Di Bello and Verheij, 2020), as well as in case-based reasoning (Liu et al., 2022a; Prakken and Ratsma, 2022). Prakken and Ratsma (2022) reconstruct explanations—and in particular contrastive explanations in the context as argument games between a proponent and opponent of an argument (i.e., a case citation for an outcome to be explained). Liu et al. (2022a) directly follow Miller (2019) and argue that a case base can be represented through a binary classifier: thus contrastive and counterfactual explanations are used to explain the outcomes of the classifier. Though valuable, those systems work on cases having the form c = (s, X, c), where *s* is a state/fact situation, *c*∈{0, 1} (the outcome favors the defendant or the plaintiff), and *X*, called the *reason* of the decision, is a subset of *s*. The structure of decisions and legal reasoning is much richer in our framework.

An interesting contribution in legal reasoning—but mainly focused on legal evidence—is Borg and Bex (2020), which develops similar notions of explanation by sufficient and necessary arguments. The idea of contrastive is also considered, but the approach is technically rather different. The authors, given the question “why *P* rather than *Q*?”, call *P* the fact and *Q* the foil (Lipton, 1990). The constrative explanation aims at making the foil explicit and considers those arguments that explain: (a) the acceptance of the fact and the non-acceptance of the foil; (b) the non-acceptance of the fact and the acceptance of the foil. Our approach provide several options that exploit the structure of argumentation theories, and which are not discussed by Borg and Bex (2020) (such as the distinction between factual and plain arguments).

### 6.3. Norm revisions and legal reasoning

As we have shown, the idea of contrastive and stable explanation require the current argumentation framework to be changed. Hence, an interesting issue is rethinking the quest for an explanation as an abductive inference, based on the revision of the given argumentation theory (Governatori and Rotolo, 2010; Governatori et al., 2019). Formally, given the argumentation theory *D*_*init*_, the revised theory *D*, and the target conclusion *ϕ*, we could formally define change operations as follows:

**Expansion**: from *D*_*init*_⊬*ϕ* to *D*⊢*ϕ*.
**Contraction**: from *D*_*init*_⊢*ϕ* to *D*⊬*ϕ*.
**Revision**: from *D*_*init*_⊢*ϕ* to *D*⊢*~*ϕ**.

The development of this intuition has to be left to future research.

Another interesting future development concerns the import of the proposed idea of explanation in legal theory. While it is well-known that the idea of explanation can be used to reconstruct causality, it is less clear how to apply it to normative reasons. It can be interesting to mention here an exponent of classical doctrine of case law, Wambaugh (1894), who stated that the identification of the ratio decidendi of a precedent starting from a particular datum—understood as part of the argumentative framework—is reduced to a procedure in which one must ask whether, by denying this datum, the court could reach the conclusion obtained. This suggests that various types of explanation can play an interesting role in case-based reasoning (Liu et al., 2022a), including the idea of conterfactual explanation (Miller, 2019), which is left as well to future research.

## 7. Conclusion

In this paper we have discussed the role of argumentation in the law, and reviewed some literature of formal models of legal argumentation. Then we have investigated the formal connection between argumentation and explanation in the law. In particular, we have proposed several definitions of an explanation in the context of formal argumentation, articulating the relations between the justification of arguments and explanations.

One basic theoretical challenge was at the core of our contribution: clarifying through formal argumentation the structure in normative reasoning of the concepts of justification and explanation. In legal theory, the focus usually is on providing a justification for legal decisions, so that the idea of an explanation only plays a secondary role. This is due to the fact that on the one hand it is assumed that legal decision-making requires strong standard of (internal) rationality, and on the other hand the notion of an explanation is usually confined to what we called causal explanation, rather than to rational reconstruction.

In this paper we took a different perspective, which is closer to how the concept of explanation has been formally developed in logic and adopted in XAI. We argued that the distinction between justification and explanation is pragmatical rather than structural. Thus we can include rational reconstructions within the scope of explanation, and have argued that such reconstructions can be extracted from justifications, to provide an account of the logic of such justification with regard to the issues at stake. Thus, we have developed various notions of explanation on top of the justification of arguments and conclusions, such as different kinds of contrastive explanations.

We have also presented the idea of stable normative explanation (Governatori et al., 2022c). The problem of determining a stable normative explanation for a certain legal conclusion means to identify a set of facts, obligations, permissions, and other normative inputs able to ensure that such a conclusion continues to hold when new facts are added to a case. This notion is interesting from a logical point of view—think about the classical idea of inference to the best explanation—but it can contribute to symbolic models for XAI for the law (consider, for instance, systems of predictive justice).

## Data availability statement

The original contributions presented in the study are included in the article/supplementary material, further inquiries can be directed to the corresponding authors.

## Author contributions

All authors contributed to the article and approved the submitted version.

## Footnotes

^1^The introduction and parts of Section 3 elaborate on materials from Prakken and Sartor (2015).

^2^In several systems other two kinds of rules are allowed: strict rules and defeaters. A strict rule is a rule in the classical sense: whenever the antecedent holds, so indisputably is the conclusion. A defeater is a rule that cannot be used to draw any conclusion, but can provide contrary evidence to complementary conclusions.

^3^This simplification does not affect the generality of the approach. Such an assumption, which can be abandoned (see Governatori et al., 2004), allows for exploring some interesting properties of explanations: see Governatori et al. (2022b) and Section 5.4.

^4^ https://www.osservatoriosullefonti.it/emergenza-covid-19/fonti-governative/decreti-del-presidente-del-consiglio-dei-ministri/2997-emcov-dpcm-elenco

^5^ https://www.legislation.gov.au/Details/C2009A00134

## Appendix: proof of Theorem 1

Proof.Rule-based grounded semantics are characterized by Defeasible Logic with ambiguity propagation (*DL_p_*) (Antoniou et al., 2001), and so we know that, given any argumentation theory *D* and for any conclusion ψ, *D*⊢*DL_p_*ψ (resp. *D*⊬*DL_p_*ψ) iff there exists an argument *A* in *GE*(*D*) such that Conc(*A*) = ψ (resp. there exists no argument *A* in *GE*(*D*) such that Conc(*A*) = ψ) under grounded semantics (Governatori et al., 2004, Theorem 3.12). Accordingly, we can resort, with some minor modifications, to the proof developed by Governatori et al. (2022b) and which is based on the proof-theoretic properties of Defeasible Logic. We show that the complement of the considered problem is NP-complete. Namely, given the argumentation theory and the normative case, the problem is to show that the case is not stable. Hence, we have to show that a superset of the explanation that does not prove the target literal exists using the proof theory described by Governatori et al. (2004). As usual, the proof consists of two parts. Given an oracle that guesses a theory where the set of facts is a superset of the one corresponding to the explanation, we can check polynomially whether this theory proves the target literal or not [which is a standard result of Defeasible Logics (Maher, 2001)]. For the second part, we provide a polynomial encoding of 3-SAT, and we demonstrate that if the theory encoding the 3-SAT instance is not stable, then the 3-SAT instance is satisfiable. A 3-SAT instance is given by

$$\overset{n}{\underset{i=1}{\wedge }}\phi_{i}$$

where $\phi_{i}=\psi_{i}^{1}\vee \psi_{i}^{2}\vee \psi_{i}^{3}$. Its encoding in Defeasible Logic is given by the argumentation theory *D* = (*R*, ∅, ∅) where *R* contains, for every clause *ϕ*_*i*_, the following rules^6^:

$$\begin{array}{l} r_{i,j}:\psi_{i}^{j}\Rightarrow \phi_{i}\;j\in \left\{1,2,3\right\} \end{array}$$

plus the two rules:

$$\begin{array}{l} r_{sat}:\phi_{1},\ldots ,\phi_{n}\Rightarrow sat\\ r_{nsat}:\Rightarrow \neg sat \end{array}$$

The encoding is polynomial in the size of the 3-SAT instance. We consider the case given by the empty set of facts and ¬*sat* as the target literal. It is immediate to verify that *D*⊢*DL_p_*¬*sat*: *r*_*nsat*_ is the only applicable rule. The set of admissible facts (see Definition 3) consists of all literals $\psi_{i}^{j}$ and $\neg \psi_{i}^{j}$. To show that is not stable we have to find a subset of admissible facts *C* such that D′=(R,C,∅)⊢DLp¬sat.^7^ For a (consistent) set of admissible facts *C*, we build the interpretation *I* as follows:

$$I(\psi_{i}^{j})=\{\begin{array}{ll} TRUE & \psi_{i}^{j}\in C\\ FALSE & \text{otherwise} \end{array}$$

We cannot show that D′⊢DLp¬sat iff $I⊧\wedge_{i=1}^{n}\phi_{i}$. To disprove ¬*sat*, the rule *r*_*sat*_ has to be applicable. This means we need to prove *ϕ*_*i*_. This implies that for each *ϕ*_*i*_ at least one of the rules *r*_*i*, 1_, *r*_*i*, 2_ and *r*_*i*, 3_ is applicable too. Consequently, one of $\psi_{i}^{1}$, $\psi_{i}^{2}$, and $\psi_{i}^{3}$ is derivable. Given there are no rules for $\psi_{i}^{j}$, $\psi_{i}^{j}$ is provable iff $\psi_{i}^{j}\in C$. Accordingly, $I\left(\psi_{i}^{j}\right)=TRUE$. Thus, for every clause we have an element in it that makes the clause true, thus *I*(*ϕ*_*i*_) = *TRUE*, for every *i* and so the 3-SAT instance is satisfiable. Conversely, when $I⊧\wedge_{i=1}^{n}\phi_{i}$, *I*⊧*ϕ*_*i*_ for every 1 ≤ *i* ≤ *n*. Thus, for each *ϕ*_*i*_, there is a $\psi_{i}^{j}$ such that $I\left(\psi_{i}^{j}\right)=TRUE$, and so $\psi_{i}^{j}\in C$. Therefore, $D^{\prime }{⊢}_{DL_{p}}\psi_{i}^{j}$, from which we derive that for every *i*, $D^{\prime }{⊢}_{DL_{p}}\phi_{i}$, making *r*_*sat*_ applicable, which implies D′⊢DLp¬sat.

Of course, the following holds as well.

Theorem 2. Given a theory *D* and an explanation by sufficient facts F relative to *D*, the problem of determining if F is not stable is NP-complete.
